# *Galleria mellonella* as an infection model to investigate virulence of *Vibrio parahaemolyticus*

**DOI:** 10.1080/21505594.2017.1384895

**Published:** 2017-11-27

**Authors:** Sariqa Wagley, Richard Borne, Jamie Harrison, Craig Baker-Austin, Donatella Ottaviani, Francesca Leoni, Varaporn Vuddhakul, Richard W. Titball

**Affiliations:** a Biosciences College of life and Environmental Sciences, University of Exeter, Exeter, Devon, EX4 S4QD, UK; b Pirbright Institute, Pirbright, Surrey, UK; c Centre for Environment, Fisheries, and Aquaculture Science, Weymouth Laboratory, Weymouth, Dorset DT4 8UB UK; d Istituto Zooprofilattico Sperimentale dell'Umbria e delle Marche, Laboratorio Nazionale di Riferimento Contaminazioni Batteriologiche dei Molluschi Bivalvi, Ancona, Italy; e Department of Microbiology, Faculty of Science, Prince of Songkla University, Hat Yai 90110, Thailand

**Keywords:** Vibrio parahaemolyticus, nudix hydrolase, MutT, Galleria mellonella

## Abstract

Non-toxigenic *V. parahaemolyticus* isolates (*tdh*^−^/*trh*^−^/T3SS2^−^) have recently been isolated from patients with gastroenteritis. In this study we report that the larvae of the wax moth (*Galleria mellonella*) are susceptible to infection by toxigenic or non-toxigenic clinical isolates of *V. parahaemolyticus*. In comparison larvae inoculated with environmental isolates of *V. parahaemolyticus* did not succumb to disease. Whole genome sequencing of clinical non-toxigenic isolates revealed the presence of a gene encoding a nudix hydrolase, identified as *mutT*. A *V. parahaemolyticus mutT* mutant was unable to kill *G. mellonella* at 24 h post inoculation, indicating a role of this gene in virulence. Our findings show that *G. mellonella* is a valuable model for investigating screening of possible virulence genes of *V. parahaemolyticus* and can provide new insights into mechanisms of virulence of atypical non-toxigenic *V. parahaemolyticus*. These findings will allow improved genetic tests for the identification of pathogenic *V. parahaemolyticus* to be developed and will have a significant impact for the scientific community.

## Introduction

*Vibrio parahaemolyticus* is a human pathogen and the leading global cause of seafood-associated gastroenteritis. This disease is characterised by watery or bloody diarrhoea, vomiting, abdominal cramps, headaches, fever and nausea,^1^ following the consumption of raw or undercooked seafoods.^2–4^ Several virulence determinants in *V. parahaemolyticus* have previously been identified including adhesins (e.g. MAM7), thermostable direct haemolysin (TDH) and TDH related haemolysin (TRH) and 2 Type Three Secretion Systems, T3SS1 and T3SS2. The multivalent adhesion molecule, MAM7 is present on the surface of *V. parahaemolyticus* and has been shown to be critical for the attachment of the bacteria to eukaryotic cells during the early stages of infection.^5^ The TDH and TRH haemolysins are cytotoxic and enterotoxic pore forming toxins. The T3SS delivers effector proteins such as VopA, VopL, VopT, VopV and VopC^6–10^ into eukaryotic cells which subsequently modulate host cell processes. T3SS1 is present in both clinical and environmental isolates of *V. parahaemolyticus* whilst T3SS2 is predominantly present in clinical isolates and is associated with pandemic strains of *V. parahaemolyticus* and large outbreaks of disease.^11^ The *tdh, trh* and the T3SS2 genes are currently considered to be the main virulence determinants for this pathogen.^12^

Recently, the isolation of clinical strains that lack the *tdh, trh* and T3SS2 genes has been reported.^13–16^ For example, in South Thailand between 2001–2010, 9–10% clinical isolates from patients were identified as non-toxigenic.^13,14^ In 2010, clinical isolates lacking *tdh, trh* and T3SS2 genes were isolated from patients affected with acute gastroenteritis in Torino, Italy as a result from the consumption of indigenously produced seafood.^15^ A number of possibilities have been proposed to explain the apparent association of non-toxigenic isolates with disease. Firstly, the ingestion of a mixed population of bacteria might allow the simultaneous growth of toxigenic and non-toxigenic bacteria and a single isolate per patient may lead to misdiagnosis of the aetiological agent.^15,17^ Secondly, it is possible that the deletion of virulence genes may have occurred during bacterial growth *in vitro* or *in vivo*. For example, it has previously been shown that a clinical *V. parahaemolyticus* isolate lacking *tdh* resulted from an insertional sequence-mediated gene deletion of a previously *tdh*-positive strain.^17,18^ Finally, non-toxigenic *V. parahaemolyticus* isolates may possess novel virulence mechanisms.

The investigation of *V. parahaemolyticus* virulence mechanisms requires a suitable infection model. Several models have previously been used including orogastric and peritoneal mouse models, orogastric infection of infant rabbits and rabbit ileal-loop models. Oro-gastric models reveal diarrhoea and enteritis and have been useful for studying intestinal colonisation and pathogenesis. *Galleria mellonella* larvae of the greater wax moth have been investigated as an infection model since the 1980's^19^ but over the past few years there has been an escalation of interest in this model and the larvae have been shown to be susceptible to a wide range of bacterial and fungal pathogens.^20,21^ In this study we investigated the utility of *G. mellonella* to identify virulence mechanisms of *V. parahaemolyticus*. This model has also allowed us to investigate mechanisms of virulence of non-toxigenic *V. parahaemolyticus* isolates, providing new insight into their clinical significance.

## Results

### The presence of non-toxigenic strains from clinical samples

We examined 10 strains of *V. parahaemolyticus*, 2 originating from the environment while the rest were clinical samples, these have all been listed in Table 1. Using PCR, we screened 6 clinical *V. parahaemolyticus* isolates previously known to be negative for virulence genes (*tdh*^−^/*trh^−^*/ T3SS2^−^) (Table 1) as well as 2 environmental strains for the species marker *toxR* gene. All of the *V. parahaemolyticus* isolates were positive for the species marker *toxR*. We then confirmed that 7 of the *V. parahaemolyticus* strains were negative for the *tdh, trh* and *T3SS2* genes. These included *V. parahaemolyticus* PSU 3384 and PSU 3565, isolated from patients in Thailand, in 2005.^13^ and T03, T08, T023 and T024 isolated from patients in Italy, in 2010.^15^ We also found one environmental isolate V13/003A from the UK which lacked these genes. The environmental strain V05/70, isolated from shellfish in Portugal, was positive for the *trh* and T3SS2β genes. The sequenced reference strain, *V. parahaemolyticus* RIMD 2210633,^22^ was positive for the *tdh* and T3SS2α genes. Clinical strain *V. parahaemolyticus* G35, isolated from a recent outbreak in Spain in 2012 and serotyped as O4:K12,^23^ was positive for *tdh, trh*, T3SS2α and T3SS2β genes.

**Table 1.** *Vibrio parahaemolyticus* strains used in this study.

Name	Type	Location	Serotype	ToxR	TDH	TRH	T3SS1	T3SS2α	T3SS2β	Survival^a^
T08 47053*	Clinical	Italy		+	–	–	+	–	–	10%
T024 47060*	Clinical	Italy		+	–	–	+	–	–	23%
T023 47066*	Clinical	Italy		+	–	–	+	–	–	NT
T03 47070	Clinical	Italy		+	–	–	+	–	–	67%
PSU 3565*	Clinical	Thailand	O3:K75	+	–	–	+	–	–	10%
PSU 3384*	Clinical	Thailand	O11:K40	+	–	–	+	–	–	NT
G35*	Clinical	Spain	O4:K12	+	+	+	+	+	+	7%
V13/003	Environmental	UK		+	–	–	+	–	–	93%
V05/070	Environmental	Portugal	O11:KUT	+	–	+	+	–	+	77%
RIMD2210633	Clinical	Japan	O3:K6	+	+	–	+	+	–	47%

*Notes. *Strains highlighted with * were used for whole genome sequencing, a = average % survival in Galleria mellonella after 24 hrs, NT = not tested.

### V. parahaemolyticus shows virulence in G. mellonella model

**Figure 1.**
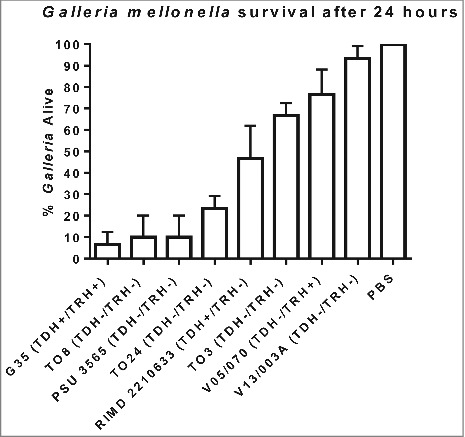
Survival of *G. mellonella* following infection with 100 CFU per larvae of *V. parahaemolyticus* strains RIMD 2210633, G35, T08, PSU 3565, T024, T03, V05/070 or V13/003A. The results shown are the means of three experiments, each using groups of 10 larvae per strain. The error bars indicate standard deviation.

We next challenged groups of 10 *G. mellonella* larvae by injection into the foreleg with 100 CFU of *V. parahaemolyticus* G35, T08, T03, T024, PSU 3565, V05/70, V13/003A or RIMD 2210633 and the results are shown in Figure 1. Approximately 20% of the larvae survived 24 h after inoculation with strains G35, T08, PSU 3565 and T024 (Statistically significant when compared to PBS using one way-ANOVA P < 0.0001). The O4:K12 serotype, which until 2012 was unique to the North West pacific region of the USA, has been shown to be the more virulent in humans resulting in a higher attack rate during outbreaks compared to other pathogenic strains.^23^ In 2012, this serotype O4:K12 was responsible for an outbreak in Spain where 51 cases were detected among the 65 people who were exposed to the pathogen which represents a 78% attack rate. We found that a serotype O4:K12 isolate (G35) was the most virulent strain in *G. mellonella* and by 24 h post challenge less than 7% of the larvae survived (Figure 1). The non-toxigenic clinical isolates (T08, T024 and PSU 3565) which lacked the *tdh, trh* and T3SS2 genes were virulent in *G. mellonella* with less than 24% survival of larvae at 24 h post challenge ((P < 0.0001). In contrast the non-toxigenic environmental isolate V13/003A, caused only 6% mortality of *G. mellonella* larvae after 24 h as seen in Figure 1 (not statistically significant). The LD50 was not determined for each of these strains but instead equivalent number of bacterial cells were used to compare their virulence in *G. mellonella.* We also found that clinical strain *V. parahaemolyticus* T03 which was non-toxigenic showed almost 65% survival rate after 24 h in the larvae model. Whilst we were unable to carry out genome sequencing on strain T03 (due to completion of project) we obtained a positive PCR result when using PCR primers designed to detect *mutT* (data not shown) in strain T03. When we tested *V. parahaemolyticus* strain RIMD2210633, a well characterised clinical TDH positive reference strain that has had its genome fully sequenced, the larvae showed 47% survival (Figure 1). We also tested for comparison V05/070 an environmental TRH positive strain which showed 77% survival in the insect model.

### Multi locus sequence analysis (MLSA)

We genome sequenced 5 non-toxigenic clinical *V. parahaemolyticus* strains (Table 1) (T08, T023, T024, PSU 3565 and PSU 3384) and one toxigenic strain (G35). MLSA, using the *recA, gyrB, pyrH and atpA* sequences, revealed that all of the *V. parahaemolyticus* strains grouped together and were distinct from closely related bacterium *Photobacterium profundum* (Figure 4). MLSA revealed that strains T023 and T024 shared more than 99% similarity and are likely clones.

### Identification of functional insertional sequences (ISVpa3)

To determine whether the clinical non-toxigenic strains T08, T023, T024, PSU 3384 and PSU 3565 were derived from previously toxigenic strains harbouring *tdh/trh* and genes for T3SS2 we searched for functional insertional sequences (ISVpa3) that surrounded the PaI-7 described by Kamruzzaman, *et al*. (2008) using BLASTn. ISVpa3 region flanks the two *tdh* sequences that can be found in *tdh*^+^ strains. We found that strain G35, had a sequence with 98% match to ISVpa3, as expected for a *tdh and trh* positive strain. Strains PSU 3384, T023 and T024 possessed DNA sequences with 43% matches to the ISVpa3. These results suggests that it is unlikely that *tdh/trh* and the genes for T3SS2 had been present in strains prior to infection. Strains T08 and PSU 3565 did not possess the ISVpa3 sequence.

### Confirming the presence/absence of classical virulence factors

We confirmed the presence or absence of known virulence factors in the genome sequences of 5 non-toxigenic *V. parahaemolyticus* strains T08, T023, T024, PSU 3565 and PSU 3384 and one toxigenic strain G35 by carrying out BLASTn searches. Table 3 shows a summary of the main virulence factors and pathogenicity islands present in Reference strain RIMD2210633 and their corresponding absence/presence in the 6 genome-sequenced strains. T3SS1 was found to be present in all strains. We found that VPaI-7 on which the genes for TDH, TRH and the T3SS2 are localised was absent in the five non-toxigenic strains we sequenced. Also absent was VPaI-1, 4, 5 and 6 from the five strains while VPaI-2 and 3 are partially present in the non-toxigenic strains.

#### Identification of a MutT/Nudix family protein

In order to identify genes unique to the clinical non-toxigenic strains we compared their genomes to available genome sequences of clinical toxigenic strains. We used Rapid Annotations using Subsystem Technology (RAST) to identify genes and found *mutT*, encoding a predicted MutT/Nudix family protein, in all five non-toxigenic strains. The predicted MutT/Nudix family protein, in all five non-toxigenic *V. parahaemolyticus* strains shared homology with nudix hydrolases from many different bacteria including *Bacillus anthracis, Legionella pneophillia* and *Lactobacillus casei*. The gene *nudA* encoding a Nudix hydrolase was shown to be an important virulence factor involved in resisting stress in *Legionella pneumophila*.^24^ Using PCR, we confirmed the presence of *mutT* in the *V. parahaemolyticus* strains that we genome sequenced. The *mutT* gene was absent in the environmental strains V13/003A and V05/070 that showed low virulence in *G. mellonella* (Figure 1).

#### A *mutT* mutant shows impaired virulence in *G. mellonella*

**Figure 2.**
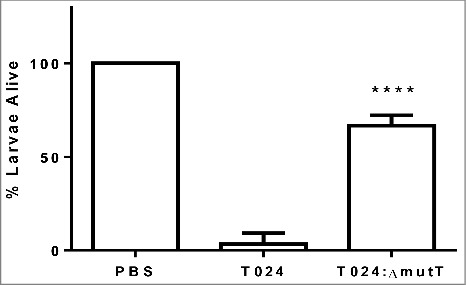
Survival of *G. mellonella* after 48h following challenge with 1–10 CFU per larvae of *V. parahaemolyticus* T24 or T24:Δ*mutT*. Groups of 10 larvae were challenged. The results shown are the means of three replicates. The error bar indicates standard deviation.

To determine if *mutT* was involved in virulence in *V. parahaemolyticus* we created an in frame deletion mutant in the strain T024, called T024*:ΔmutT*. Groups of 10 *G. mellonella* larvae were challenged by injection into the foreleg with 1–10 CFU of *V. parahaemolyticus* T024 or T024*:ΔmutT* (Fig. 2). Survival at 48 h post challenge was observed. Following challenge with *V. parahaemolyticus* T024*:ΔmutT*, 67% of infected larvae survived compared to 3% of larvae infected with wild-type (P < 0.0001). These data demonstrates that *mutT* plays a role in virulence in *G. mellonella*.

#### A *mutT* mutant shows reduced colony size

**Figure 3.**
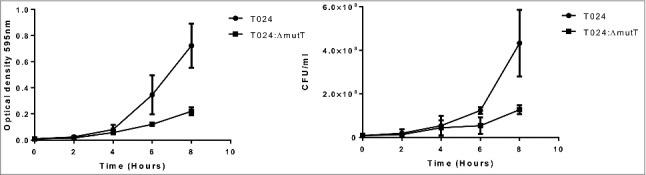
Growth of T024 or T024:Δ*mutT* at 30°C in minimal media measured by optical density (left panel) or CFU (right panel). At 8 hr there is significant difference between the wildtype and the T024:*ΔmutT* (determined by a paired T test *P* < 0.05) while no significant difference is seen with the cell counts.

The deletion mutant of *nudA* in *L. pneumophila* grew slowly in liquid media and on solid media and had a smaller colony size than its parent strain.^24^ In this study we chose minimal media which contained minimal nutrients possible to assess growth and colony size of the wild type and mutant strains. Wild type and mutant strains were preadapted to the minimal media by growing overnight in minimal media and then this was used to inoculate fresh media with equal numbers of the parent or mutant and incubated at 30°C. The wildtype strain T024 had a higher OD than the T024*:ΔmutT* at 8 h, while the CFU counts were similar (Figure 3). Although we did not examine the cells microscopically *V. parahaemolyticus* T024:Δ*mutT* grew smaller in colony size than the T024 wild type strain on agar.

**Figure 4.**
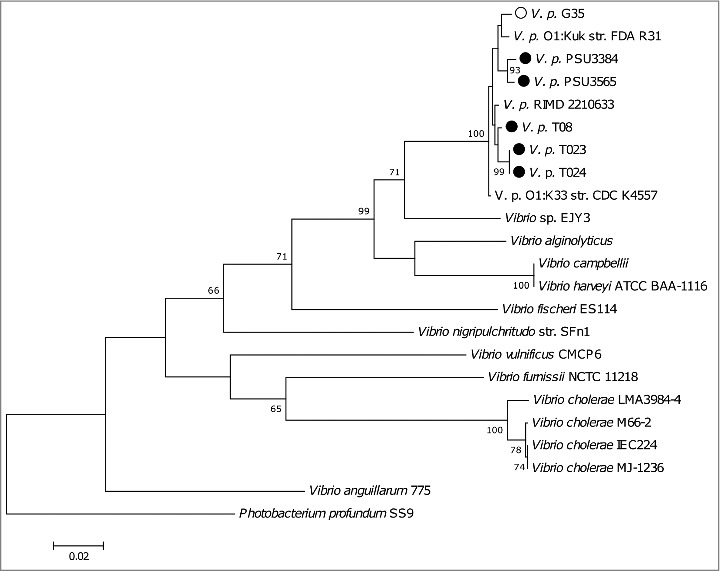
A phylogenetic tree compiled from *recA, gyrB, pyrH and atpA* sequences from sequenced *V. parahaemolyticus* strains including the reference strain RIMD 2210633.

## Discussion

In this study we report for this first time that *G. mellonella* is susceptible to infection with *V. parahaemolyticus*. Previous studies have used *G. mellonella* to identify virulence associated genes in a range of pathogens including *Pseudomonas aeruginosa*.^25^ *Enterococcus faecalis*,^26^ *Staphylococcus aureus*^27^ *Yersinia pseudotuberculosis*^28^ and *Campylobacter jejuni*.^29^ *G. mellonella* possess haemocytes that share a high degree of structural and functional similarity with mammalian phagocytic cells. The cellular response of *G. mellonella* to infection involves haemocytes that ingest bacteria and generate bactericidal products such as superoxide via a respiratory burst.^30^ Haemocyte nodulation results in melanisation through the action of phenoloxidase.^31,32^ The degree of melanisation in infected larvae results in a mottled grey to uniform black appearance, depending on the infecting bacteria. Insect larvae can be infected at 37°C, ensuring expression of temperature regulated virulence genes. Moreover, a defined infection site and the ability to challenge larvae with exact doses allows the virulence of strains to be compared. Our study shows that virulence in *G. mellonella* appears to correlate with the ability of isolates to cause disease in humans and demonstrates the value of *G. mellonella* for the identification of genes associated with virulence in *V. parahaemolyticus*. Current infection models to study *V. parahaemolyticus* infections include mammals such as mouse and infant rabbits. Although these mammalian models allow the study of virulence mechanisms, they are expensive, require specialist training, and need ethical approval before studies can be carried out. The use of a non-mammalian model to study *V. parahaemolyticus* such as larvae allows high throughput screening of a large number of *V. parahaemolyticus* strains at low costs. In this study we used *G. mellonella* as an initial screen to determine virulence of *V. parahaemolyticus* and reduce the reliance on experimental mammals. The availability of this infection model will be of value to others investigating mechanisms of virulence of *V. parahaemolyticus*. Furthermore, the simplicity of this insect model over other non-mammalian models will help the scientific community to better understand and investigate pathogenic traits of *V. parahaemolyticus* strains.

Previous work has attributed the ability of *V. parahaemolyticus* to cause disease in humans to the presence of genes encoding the TDH and/or TRH haemolysins and to the T3SS2 genes. Environmental strains of *V. parahaemolyticus* carry these virulence genes less frequently and are thought not able to illicit disease in humans.^33^ However, there is growing evidence that strains lacking these genes can cause disease. For example, at Hat Yai Hospital in South Thailand 10% of *V. parahaemolyticus* infections are attributed to non-toxigenic strains. We showed in this study that a set of non-toxigenic *V. parahaemolyticus* strains were phylogenetically similar to other *V. parahaemolyticus* strains and did not carry the conventional virulence genes associated with disease such as *tdh/trh* and the genes for the T3SS2. We have shown that *G. mellonella* (wax moth) larvae are susceptible to a lethal infection with toxigenic and non- toxigenic clinical strains of *V. parahaemolyticus*. However, we noted some important differences in the pathogenesis of disease. Signs of infection after challenge with toxigenic strains occurred within a short period of time (<2 h) and included melanisation, reduced motility/movement, faecal staining and death. When clinical non-toxigenic *V. parahaemolyticus* strains were injected into larvae at comparable doses they caused disease and death in the larvae, but the rapid onset of melanisation was not observed. These findings suggest that the rapid effects seen after dosing with toxigenic strains may be a consequence of the direct action of the TDH or TRH toxins on the larvae.

Nudix hydrolases have been shown to be involved in virulence of a number of different pathogens.^24,34,35^ For example, in *Legionella pneumophila* NudA is an important stress prevention factor and *nudA* mutants showed they were outcompeted in macrophage studies by fourfold by the parent in competition studies.^24^ Further competition studies in guinea pigs also showed that the *nudA* mutant of *Legionella pneumophila* was outcompeted by its parent in both lung and spleen.^24^ In this study we identified *mutT*, a nudix family protein present in the non-toxigenic strains of *V. parahaemolyticus* and suggest it may have a role in establishment of disease in strain T024. The reduced colony size observed of T024*:ΔmutT* did not lead to an altered growth rate. We were unable to construct a complemented strain of *mutT* in T024 to show that there were no polar effects that may have occurred from genetic manipulation in the T024 *mutT* mutant. However, during our initial screening of *mutT* mutants we found that multiple colonies gave comparable results in growth curves and initial screening in the *G. mellonella* model. Further studies where *mutT* mutants constructed in strains PSU3565 and T08 may help support that mutT is a general virulence factor in *V. parahaemolyticus* species. Nudix hydrolases are able to hydrolyse organic pyrophosphates, such as nucleoside diphosphates and triphosphates, as well as nucleotide sugars. Nudix hydrolase substrates or alarmones are hypothesised to be produced in high concentration in response to heat shock or oxidative stress. Further investigation is required to establish the function and role of *mutT* in *V. parahaemolyticus* strains that harbour this gene and the role it may play in establishing disease. Analysis of freely available complete genomes on NCBI (N = 17) found the *mutT* gene to be present in 3 environmental strains of *V. parahaemolyticus* (FORC 006, FORC 023 and FORC 022) that did not harbour any genes for *tdh/trh* and T3SS2. Thus, it may be important to determine the distribution of this gene in a broader range of clinical and environmental isolates of *V. parahaemolyticus*, to establish the role of *mutT* in virulence and to establish whether this gene is a useful marker of non-toxigenic clinical strains of *V. parahaemolyticus*.

## Methods and materials

### Strains and cultures used

Bacterial strains used in this study are shown in Table 1. *V. parahaemolyticus* strains were initially cultured aerobically onto selective media Thiosulphate Citrate Bile Sucrose (TCBS) agar (Oxoid) at 37°C for 24 h to check for contamination. For enumeration of colony counts we used Marine Agar (Conda lab, India) at 30°C for 24 h and for routine subculturing and growth Luria-Bertani (LB) agar at 37°C for 18 h was used. For growth curves, wildtype T024 and T024:*ΔmutT* were grown in minimal media with aeration at 30°C and optical readings and colony counts were carried out on Marine agar at 0, 2, 4, 6 and 8 h. For virulence assays the *V. parahaemolyticus* strains were grown in Marine Broth (Conda Lab, India) at 37°C.

### PCR identification of genes

Bacterial cells were harvested and genomic DNA was extracted using Wizard Genomic DNA Purification Kit (Promega A1125). A *ToxR* targeted PCR (Table 2) was used to confirm the identification of *V. parahaemolyticus* using primers described previously.^36^ The *tdh* and *trh* genes were detected using previously described primers (Table 2).^37^ Multiplex PCRs were carried out to detect the T3SS1 and T3SS2α genes using primers described previously by Noriea et al.^16^ while T3SS2β genes were detected using primers described previously by Okada et al.^38^ PCR was used to confirm the presence of the *mutT* gene in *V. parahaemolyticus* using the primers 5′-ATCCCCGGCGTTGCGGGTGTCATT-3′ and 5′-GCCAACGAAGTACGCTGAATCATTTTC-3′. The PCR amplification cycle for amplification of *mutT* consisted of 15 minutes at 96°C, followed by 30 cycles of 1 minute at 94°C, 1.5 minutes at 60.5°C and 1.5 minutes at 72°C, and finally with a single extension time of 7 minutes at 72°C. All PCR reactions described above were carried out using Hot Start Taq (Qiagen) and set up following the manufactures instructions. PCR reactions were run on a 1.5% agarose gel stained with SYBR Safe DNA stain and visualised using Chemidoc imaging system equipped with QuantityOne software (BioRad).

**Table 2.** Oligonucleotide primers used in this study.

Target	Name	Sequence	Reference
ToxR	ToxR Fw	GTCTTCTGACGCAATCGTTG	36
	ToxR Rv	ATACGAGTGGTTGCTGTCATG	
TDH	TDH fw	CCACTACCACTCTCATATGC	37
	TDH Rv	GGTACTAAATGGCTGACATC	
TRH	TRH Fw	GGCTCAAAATGGTTAAGCG	37
	TRH Rv	CATTTCCGCTCTCATATGC	
T3SS1	VP1670F	ACCGATTACTCAAGGCGATG	16
	VP1670R	TACGTTGTTGGCGTGATTGT	
	VP1686F	CAAAAGCGATCACAAAAGCA	
	VP1686R	AGCGACTTAACGGCATCATC	
	VP1689F	AAGGTTGGCAAAAAGCGTTA	
	VP1689R	GCTCTTCAACGAGCCAAGAG	
	VP1694F	ACGATGCGACCAACAGTGTA	
	VP1694R	TTTTAATTGCATCGGTGACG	
T3SS2α	VPA1327F	TGGCGAAAGAGCCATTAGAT	16
	VPA1327R	TCAACTCCAAATTCGCCTTC	
	VPA1335F	ATGTAACGGCGGCTAGCTTA	
	VPA1335R	CAAACTGTGTCAGTAGCACCA	
	VPA1339F	GATTCGCGGAACTCAAGAAG	
	VPA1339R	CTTGTCCGAGATCAACGTCA	
	VPA1362F	CTGCAGGTATCGCATCTTCA	
	VPA1362R	TTAGAACCAACCGACGAAGC	
T3SS2β	Beta_vscC2_F	GTACTTTGCTGTCTAACC	38
	Beta_vscC2_R	CTTACTCTTAACTTCCGACG	
	Beta_vscS2_F	TTGATGTTGTTTCGGCTAGC	
	Beta_vscS2_R	CCACCGCCGAACTCGGCTAACAAG	
	Beta_vopB2_F	GAGCCTGTTGCTCTATGGAGCCAGG	
	Beta_vopB2_R	CGACACAGAACGCAATGCTTGCTCG	
	Beta_vopC_F	AACCAACTTGCGACTAAATC	
	Beta_vopC_R	TCCCGACAGTTTTTCTGCAC	
*MutT* flank 1	fw	ACAGAGCTCATATGCTGTATGGAAAT	This study
*MutT* flank 1	Rv	GCTCTAGACAATTTTCCTCCTTGAAA	This study
*MutT* flank 2	Fw	TGGCTCTAGATAGAAAGCTAACAAATT	This study
*MutT* flank 2	Rv	TTGCATGCATTCATTTTGTCATCGC	This study
*ΔMutT* confirmation	Fw	GTAGAAAATTCGAGGCTTTGTTGTTTTGC	This study
*ΔMutT* confirmation	Rv	CGAGCTTCAACATAAACAAGTACATGC	This study

**Table 3.** Presence or absence of chromosomal regions in 5 non-toxigenic strains (T08, T023, T024, PSU 3565 and PSU 3384) and 1 toxigenic strain (G35) when compared to reference strain RIMD2210633 and their corresponding absence/presence in the 6 genome-sequenced strains.

ORFs	Region Type	T08	T023	T024	PSU 3565	PSU 3384	G35	Reference
Chromosome 1								
VP0218 – VP0234	LPS	°	°	°	°	°	°	
VP0380 – VP0403	VPaI-1	°	°	°	°	°	°	
VP0634 – VP0643	VPaI-2	P	P	P	P	P	P	
VP1071 – VP1095	VPaI-3	P	P	P	P	P	P	
VP1386 – VP1414	T6SS1	•	°	°	°	°	°	
VP1549 – VP1590	phage f237	P	P	P	P	P	P	
VP1658 – VP1702	T3SS-1	•	•	•	•	•	•	
VP2131 – VP2144	VPaI-4	°	°	°	°	°	°	
VP2900 – VP2910	VPaI-5	°	°	°	°	°	°	
Chromosome 2								
VPA1253 – VPA1270	VPaI-6	°	°	°	°	°	°	
VPA1025 – VPA1046	TSS2	•	•	•	•	•	•	
VPA1312 – VPA1395	VPaI-7 (T3SS-2)	°	°	°	°	°	P	
VPA1403 – VPA1412	CPS	•	•	•	•	•	•	
VPA1559 – VPA1583	Multidrug efflux	P	P	P	P	P	•	

*Notes. *•: Present.

°: Absent.

P: Partially present.

### Infection of galleria mellonella larvae

*G. mellonella* larvae were purchased from Livefoods UK Ltd (Rooks Bridge, Somerset, UK) or TruLarv™ (Biosystems Technology, Exeter, Devon, UK).^28,39^ Larvae weighing between 0.2 – 0.3 g were chosen for experiments. For each experiment a total of 10 larvae were used per strain to be tested. The larvae were infected by micro-injection (Hamilton Ltd) into the right foremost proleg with 100 CFU of *V. parahaemolyticus* in 10 µl volume which had been grown in Marine Broth at 37°C and washed twice in PBS. Bacterial cell counts were carried out by plating serial dilutions of the inoculum onto Marine agar. For control purposes 10 larvae were inoculated with PBS and a further 10 were left uninoculated. The larvae were incubated at 37°C and survival was recorded for all strains after 24 h. An additional 48 h time point was needed for experiment with the T024:Δ*mutT*. Larvae were scored as dead when they ceased moving, and failed to respond when gently manipulated with a pipette tip. Observation findings were also recorded if larvae colour changed from their normal pale cream coloration to brown or black indicative of melanisation.

### Genome sequencing, assembly and annotation

Seven *V. parahaemolyticus* strains (Table 1) were genome sequenced. Genomic DNA (gDNA) was extracted using the Wizard Genomic DNA Purification Kit (Promega). Library prep was carried out by Exeter DNA sequencing service. In brief, DNA was concentrated using GeneRead kit (Lot No. 145025210) and end repair and adenylation of fragments was carried using NEXTflex Rapid DNAseq kit (#5144–02) according to manufactures instructions. Purification and concentration of PCR amplified library was carried out according to GeneRead kit instructions. Genome wide sequence data was generated using an Illumina Hiseq 2500 platform. Raw sequencing reads were screened for contamination and PCR duplicates and quality filtered using fastq-mcf.^48^ This filtered dataset was then assembled de-novo using velvet and velvet optimiser^40^ (final hash lengths and coverage cut offs can be found in Supplementary Data Table 1b). De-novo assemblies were annotated using Rapid Annotation using Subsystems Technology (RAST).^41,42^ Assembly metrics were generated using the Quast package.^43^

### Comparative genomics

To assess the presence or absence of genes within each of the strains, the sequencing reads were aligned against a “pan genome” consisting of all the genes annotated in each of the experimental strains. The gene sequences were clustered using UCLUST^44^ and one sequence was selected (by length) from each orthologous cluster. Trimmed sequencing reads were then aligned to this “pan-genome” using BOWTIE2^45^ to assess the presence of each gene in each strain. The benefit of this approach is that it reduces the effect of poor assemblies on the comparative genomic analysis. It is only required for each gene to have been assembled once for it to appear in the “pan-genome” and aligning sequencing reads means even if the gene was not assembled correctly in any of the other strains, the reads will align to the target sequence from any of those strains in which it appears.

### Multi locus sequence analysis (MLSA)

Multi locus sequence analysis (MLSA) was conducted on *V. parahaemolyticus* sequenced strains and genomes in the NCBI genomes database (July 2017). The MLSA was conducted using 4 conserved housekeeping genes (*gryB, pyrH, recA, atpA*). These genes were extracted from the genome sequences using blast and in house scripts to parse and extract results. The MLSA sequences were aligned using MUSCLE^46^ and data was converted into a phylogenetic tree using MEGA 7^47^ with a bootstrap value of 100 and using *Photobacterium profundum* as the root of the tree.

### mutT mutant construction

DNA fragments (500bp) including upstream and downstream regions of *mutT* and flanked by *SacI* and *SphI* restriction enzymes were created using PCR and ligation. The DNA fragment was cloned into plasmid pDM4 via the *SacI* and *Sph*I sites. The presence of the cloned DNA was confirmed by PCR using primers described in Table 2 (*mutT* flank 1 and 2). The plasmid pDM4-*mutT* was maintained in *E. coli* GT115 cells and selected on LB agar containing 50 µg/ml chloramphenicol.

### Conjugation of V. parahaemolyticus T024

One ml of an overnight culture of the recombinant *E.coli* GT115 pdm4-*mutT* (donor strain), *E.coli* pKR2013 (helper strain) and the *V. parahaemolyticus* T024 (recipient strain) were centrifuged for 2 minutes. Supernatants were discarded and the pellets re-suspended in 0.5 ml LB medium. A 10 µl aliquot was pipetted onto a sterile nitrocellulose membrane on LB agar plate, either individually or with donor, helper and recipient mixed together (ratio 1:1:4), and incubated overnight at 37°C. The cells were then re-suspended in 1ml sterile PBS. Aliquots of 100 µl were plated onto LB agar plates supplemented with 100 µg/ml chloramphenicol and incubated overnight at 37ºC. Colony growth was scraped off using a sterile 10 µl loop and re-suspending in 1ml of sterile PBS and aliquots of 100 µl were plated onto TCBS plate's supplemented with 50 µg/ml chloramphenicol. After incubation at 37ºC for 5–7 days, colonies were transferred onto fresh LB plates containing chloramphenicol. The transconjugants were grown in LB broth without supplementation overnight, serially diluted in PBS and plated onto salt free LB agar containing 10% (w/v) sucrose. The plates were incubated at 24ºC for 2 to 5 days and colonies screened for chloramphenicol sensitivity and on TCBS agar. In order to confirm that chloramphenicol sensitive colonies contained the desired mutation, PCR was carried out using *ΔMutT* confirmation primers (Table 2). The deletion mutant (T024:Δ*mutT*) was used in our subsequent experiments.

## Statistical analyses

Analysis was carried out on the differences between average survivals of larvae when infected with different strains of *V. parahaemolyticus*. These values were tested for significance by performing a one way ANOVA comparing each of the strains to PBS and were performed using the GraphPad Prism software (GraphPad Software, San Diego California USA).

## Supplementary Material

KVIR_S_1384895.zip
