# Supplementary material for: Galleria mellonella as an infection model to investigate virulence of Vibrio parahaemolyticus
Source: Virulence. 2017 Nov 27;9(1):197–207. doi: 10.1080/21505594.2017.1384895 (PMC5801645; doi:10.1080/21505594.2017.1384895)
Supplement: KVIR_S_1384895.zip [file kvir-09-01-1384895-s001.zip › KVIR_S_1384895.docx]

| **Strain** | **Sequencing platform** | **No. of Reads** | **Read Length (bp)** | **GC Content (%)** |
| --- | --- | --- | --- | --- |
| G35 | Illumina HiSeq 2500 | 9,965,794 | 100 | 44 |
| PSU 3384 | Illumina HiSeq 2500 | 5,667,196 | 100 | 44 |
| PSU 3565 | Illumina HiSeq 2500 | 25,428,186 | 150 | 45 |
| T024 47060 | Illumina HiSeq 2500 | 11,187,082 | 100 | 44 |
| T08 47053 | Illumina HiSeq 2500 | 13,681,284 | 100 | 44 |
| T023 47066 | Illumina HiSeq 2500 | 8,765,728 | 100 | 44 |

| **Strain** | **Velvet hash value** | **Total number of contigs** | **N50** | **Length of longest scaffold** | **Total bases in scaffolds** | **Number of scaffolds > 1k** | **Total bases in scaffolds > 1k** |
| --- | --- | --- | --- | --- | --- | --- | --- |
| G35 | 83 | 89 | 774335 | 1186918 | 5199826 | 38 | 5183081 |
| PSU 3384 | 75 | 127 | 722793 | 979246 | 5039776 | 49 | 5012035 |
| PSU 3565 | 135 | 92 | 508828 | 1387537 | 5145403 | 36 | 5123102 |
| T024 47060 | 87 | 62 | 1361318 | 1762907 | 4978343 | 17 | 4965863 |
| T08 47053 | 87 | 89 | 519701 | 1892210 | 5170386 | 34 | 5150347 |
| T023 47066 | 83 | 68 | 867403 | 1762579 | 4977140 | 20 | 4963161 |

Supplementary Table 1a and b: Genome information on sequenced strains. 1a shows raw sequencing data information while 1b shows assembly statistics and quality metrics.
